# Crosstalk between Ca^2+^ signaling and mitochondrial H_2_O_2_ is required for rotenone inhibition of mTOR signaling pathway leading to neuronal apoptosis

**DOI:** 10.18632/oncotarget.7183

**Published:** 2016-02-03

**Authors:** Chunxiao Liu, Yangjing Ye, Qian Zhou, Ruijie Zhang, Hai Zhang, Wen Liu, Chong Xu, Lei Liu, Shile Huang, Long Chen

**Affiliations:** ^1^ Jiangsu Key Laboratory for Molecular and Medical Biotechnology, Jiangsu Key Laboratory for Microbes and Functional Genomics, College of Life Sciences, Nanjing Normal University, Nanjing, PR China; ^2^ Department of Biochemistry and Molecular Biology, Louisiana State University Health Sciences Center, Shreveport, LA, USA; ^3^ Feist-Weiller Cancer Center, Louisiana State University Health Sciences Center, Shreveport, LA, USA

**Keywords:** rotenone, apoptosis, calcium ion, CaMKII, H_2_O_2_, mTOR, Pathology

## Abstract

Rotenone, a neurotoxic pesticide, induces loss of dopaminergic neurons related to Parkinson's disease. Previous studies have shown that rotenone induces neuronal apoptosis partly by triggering hydrogen peroxide (H_2_O_2_)-dependent suppression of mTOR pathway. However, the underlying mechanism is not fully understood. Here, we show that rotenone elevates intracellular free calcium ion ([Ca^2+^]_i_) level, and activates CaMKII, resulting in inhibition of mTOR signaling and induction of neuronal apoptosis. Chelating [Ca^2+^]_i_ with BAPTA/AM, preventing extracellular Ca^2+^ influx using EGTA, inhibiting CaMKII with KN93, or silencing CaMKII significantly attenuated rotenone-induced H_2_O_2_ production, mTOR inhibition, and cell death. Interestingly, using TTFA, antimycin A, catalase or Mito-TEMPO, we found that rotenone-induced mitochondrial H_2_O_2_ also in turn elevated [Ca^2+^]_i_ level, thereby stimulating CaMKII, leading to inhibition of mTOR pathway and induction of neuronal apoptosis. Expression of wild type mTOR or constitutively active S6K1, or silencing 4E-BP1 strengthened the inhibitory effects of catalase, Mito-TEMPO, BAPTA/AM or EGTA on rotenone-induced [Ca^2+^]_i_ elevation, CaMKII phosphorylation and neuronal apoptosis. Together, the results indicate that the crosstalk between Ca^2+^ signaling and mitochondrial H_2_O_2_ is required for rotenone inhibition of mTOR-mediated S6K1 and 4E-BP1 pathways. Our findings suggest that how to control over-elevation of intracellular Ca^2+^ and overproduction of mitochondrial H_2_O_2_ may be a new approach to deal with the neurotoxicity of rotenone.

## INTRODUCTION

Rotenone, a well-known neurotoxic pesticide, is highly lipophilic and easily traverses the blood-brain barrier and cellular membranes without depending upon dopamine transporters for access to the cytoplasm of neurons [1, 2]. Once inside cells, rotenone impedes mitochondrial function leading to intracellular oxidative stress [3, 4]. Rotenone induces apoptosis by increasing generation of mitochondrial reactive oxygen species (ROS) in HL-60 and other cells [5]. Mitochondrial enzyme deficiency in rat brain is correlated with the generation of free radicals post administration of rotenone [6]. Mounting studies have demonstrated that rotenone contributes to decreased ATP synthesis, mitochondrial depolarization, and ROS generation *via* inhibiting mitochondrial respiratory chain complex I [3, 4]. Excessive ROS in turn will further inhibit complex I [7]. The vicious cycle eventually causes apoptosis of dopaminergic neurons, leading to Parkinson's disease (PD) [7-14]. Thus, rotenone is a possible etiological factor in PD. However, the molecular mechanism underlying the neurotoxicity of rotenone is still not fully understood.

Calcium ion (Ca^2+^) is important for many cellular events, such as proliferation/growth, differentiation, development and cell death [15]. When properly controlled, Ca^2+^ fluxes across the plasma membrane and between intracellular compartments play critical roles in fundamental functions of neurons, including the regulation of neurite outgrowth and synaptogenesis, synaptic transmission and plasticity, and cell survival [16]. However, disturbances in cellular Ca^2+^ homeostasis cause synaptic dysfunction, impaired plasticity and neuronal degeneration [16-19]. Especially, abnormally high levels of intracellular free Ca^2+^ ([Ca^2+^]_i_) induces overproduction of free radicals such as ROS, which can activate stress cascades, resulting in apoptosis [20, 21]. In turn, excessive or sustained ROS can also exacerbate Ca^2+^ overload and sensitize the bioactivity of Ca^2+^ [20, 22, 23]. The interconnection between Ca^2+^ and ROS alters the structures and functions of cellular proteins, and also activates or inhibits related signaling pathways, leading to neuronal apoptosis [20, 24-27].

Mammalian/mechanistic target of rapamycin (mTOR), a serine/threonine (Ser/Thr) protein kinase, regulates differentiation, development and survival in neurons [28-30]. Thus, mTOR exerts a crucial role in synaptic plasticity, learning and memory, and food uptake in adult brain [28-30]. Increasing evidence reveals that mTOR could be activated or inhibited depending on the pathologic status of the nervous system, e.g. brain tumors, tuberous sclerosis, cortical dysplasia and neurodegenerative diseases such as PD, Alzheimer's disease (AD), and Huntington's disease (HD) [28, 30]. Our group has observed that cadmium, a heavy metal polluted in the environment, induces neuronal cell death by [Ca^2+^]_i_- and/or ROS-dependent activation of mTOR signaling [31-34], whereas hydrogen peroxide (H_2_O_2_), a major radical of ROS, elicits neuronal cell death *via* suppression of mTOR pathway [35]. Recently, we have also found that rotenone evokes neuronal apoptosis *via* H_2_O_2_-dependent inhibition of mTOR-mediated phosphorylation of ribosomal p70 S6 kinase (S6K1) and eukaryotic initiation factor 4E (eIF4E)-binding protein 1 (4E-BP1) [14, 36]. Intracellular Ca^2+^ elevation is a major factor for rotenone-induced apoptosis in neuronal cells [37]. Hence, in this study, we investigated whether rotenone induces apoptosis by Ca^2+^/ROS-dependent inhibition of mTOR pathway.

## RESULTS

### Rotenone-induced neuronal apoptosis is associated with its induction of [Ca^2+^]_i_ elevation

**Figure 1:**
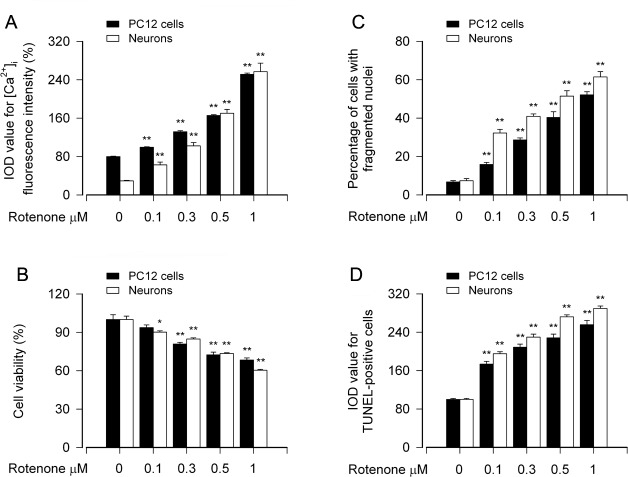
Rotenone-induced [Ca^2+^]_i_ elevation is associated with cell viability reduction and apoptosis in neuronal cells PC12 cells and primary neurons were treated with rotenone (0-1 μM) for 24 h. [Ca^2+^]_i_ fluorescence intensity was imaged and quantified using an intracellular Ca^2+^ indicator dye Fluo-3/AM (A). Cell viability was determined by the MTS assay (B) and cell apoptosis was assayed using DAPI and TUNEL staining (C, D). **A.**-**D.** Rotenone concentration-dependently elicited [Ca^2+^]_i_ elevation (A), and induced viability reduction (B) and apoptosis (C, D) in PC12 cells and primary neurons. Results are presented as mean ± SE (*n* = 5). *P < 0.05, ^**^P < 0.01, difference with control group.

Increased [Ca^2+^]_i_ levels have been documented in many experimental models of apoptosis [37-39]. To understand how Ca^2+^ signaling participates in rotenone-induced neuronal apoptosis, first of all, we investigated the relationship between the [Ca^2+^]_i_ level and the apoptosis in our neuronal cell models treated with rotenone. After PC12 cells and mouse primary neurons were treated with 0-1 μM rotenone for 24 h, [Ca^2+^]_i_ was measured by using an intracellular Ca^2+^ indicator dye, Fluo-3/AM. We found that rotenone elicited strong [Ca^2+^]_i_ fluorescence (in green) (Figure S1A), and the intensity of the fluorescence was rotenone concentration-dependent (Figure 1A). Concurrently, rotenone decreased cell viability (Figure 1B), and increased nuclear fragmentation and condensation (arrows), a hallmark of apoptosis [40], as well as TUNEL-positive cells (in green) in PC12 cells and primary neurons (Figure S1B, Figure 1C and 1D), respectively. Besides, treatment with rotenone for 24 h induced robust cleavages of caspase-3 and poly (ADP-ribose) polymerase (PARP) in the cells (data not shown). Collectively, these data imply that rotenone-induced neuronal apoptosis is associated with the induction of [Ca^2+^]_i_ elevation.

### Rotenone elicits neuronal apoptosis *via* Ca^2+^-mediated inhibition of mTOR pathway

**Figure 2:**
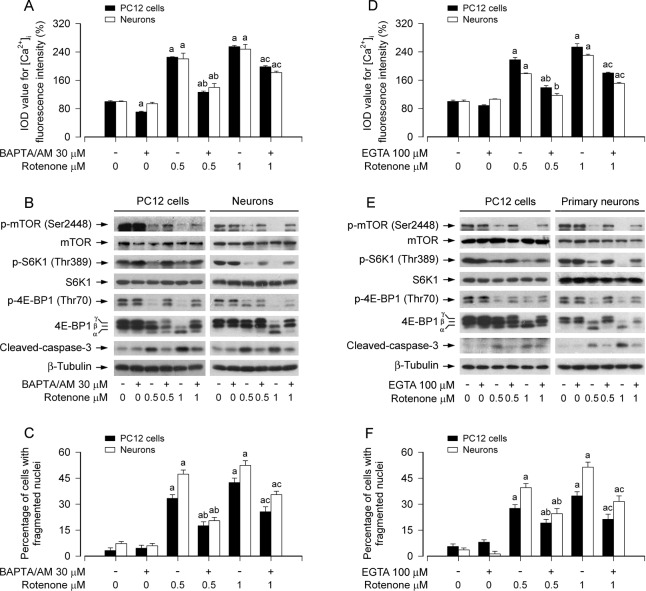
Rotenone elicits neuronal apoptosis *via* Ca^2+^-mediated inhibition of mTOR pathway PC12 cells and primary neurons were pretreated with/without BAPTA/AM (30 μM) or EGTA (100 μM) for 1 h and then exposed to rotenone (0.5 and 1 μM) for 24 h. [Ca^2+^]_i_ fluorescence intensity was imaged and quantified using an intracellular Ca^2+^ indicator dye Fluo-3/AM (A, D), Total cell lysates were subjected to Western blotting using indicated antibodies (B, E). The blots were probed for β-tubulin as a loading control. Similar results were observed in at least three independent experiments. Cell apoptosis was assayed using DAPI staining (C, F). **A.**-**F.** Pretreatment with BAPTA/AM or EGTA significantly attenuated elevation of [Ca^2+^]_i_ level (A, D) and inhibition of phosphorylation of mTOR, S6K1 and 4E-BP1 (B, E) in PC12 cells and primary neurons induced by rotenone, and obviously recused the cells from rotenone-induced cleaved-caspase-3 (B, E) and apoptosis (C, F). Results are presented as mean ± SE (*n* = 5). ^a^*P* < 0.05, difference with control group; ^b^*P* < 0.05, difference with 0.5 μM rotenone group; ^c^*P* < 0.05, difference with 1 μM rotenone group.

Our recent studies have shown that rotenone induces neuronal apoptosis by inhibiting mTOR pathway [36]. Having observed that rotenone-elevated [Ca^2+^]_i_ is involved in neuronal apoptosis (Figure 1), next we asked whether the elevation of [Ca^2+^]_i_ is responsible for rotenone-induced inhibition of mTOR signaling and apoptosis of neuronal cells. To this end, PC12 cells and primary neurons were treated with/without rotenone (0.5 and 1 μM) for 24 h following pre-incubation with/without 1,2-bis (2-amino-phenoxy)ethane-N,N,N,N-tetraacetic acid (BAPTA/AM, 30 μM), an intracellular Ca^2+^ chelator, for 1 h. We found that BAPTA/AM significantly attenuated rotenone-triggered [Ca^2+^]_i_ elevation (Figure 2A) and substantially prevented rotenone from inhibiting the phosphorylation of mTOR, S6K1 and 4E-BP1 (Figure 2B). Rotenone-induced cleavage of caspase-3 was also remarkably inhibited by BAPTA/AM (Figure 2B). In line with this, BAPTA/AM markedly prevented rotenone-induced cell viability reduction (Figure S2A), nuclear fragmentation and condensation (Figure 2C) in PC12 cells and primary neurons as well. The results suggest that rotenone-elevated [Ca^2+^]_i_ contributes to inhibition of mTOR pathway and induction of neuronal apoptosis.

To further define the role of rotenone-elevated [Ca^2+^]_i_ in suppressing mTOR pathway, we extended our studies using ethylene glycol tetra-acetic acid (EGTA, 100 μM), an extracellular Ca^2+^ chelator. The results showed that inhibition of extracellular Ca^2+^ influx by EGTA profoundly prevented rotenone-induced elevation of [Ca^2+^]_i_ (Figure 2D) and inhibition of phosphorylation of mTOR, S6K1 and 4E-BP1 (Figure 2E) in PC12 cells and primary neurons. Furthermore, EGTA also partially blocked rotenone-induced cleavage of caspase-3 (Figure 2E), viability reduction (Figure S2B) and apoptosis (Figure 2F) in the cells. The results indicate that rotenone-induced extracellular Ca^2+^ influx is involved in inhibition of mTOR pathway leading to neuronal apoptosis. Our findings strongly support the notion that rotenone elicits neuronal apoptosis via Ca^2+^-mediated inhibition of mTOR pathway.

### Rotenone elicits Ca^2+^-dependent CaMKII phosphorylation leading to inhibition of mTOR pathway and neuronal apoptosis

**Figure 3:**
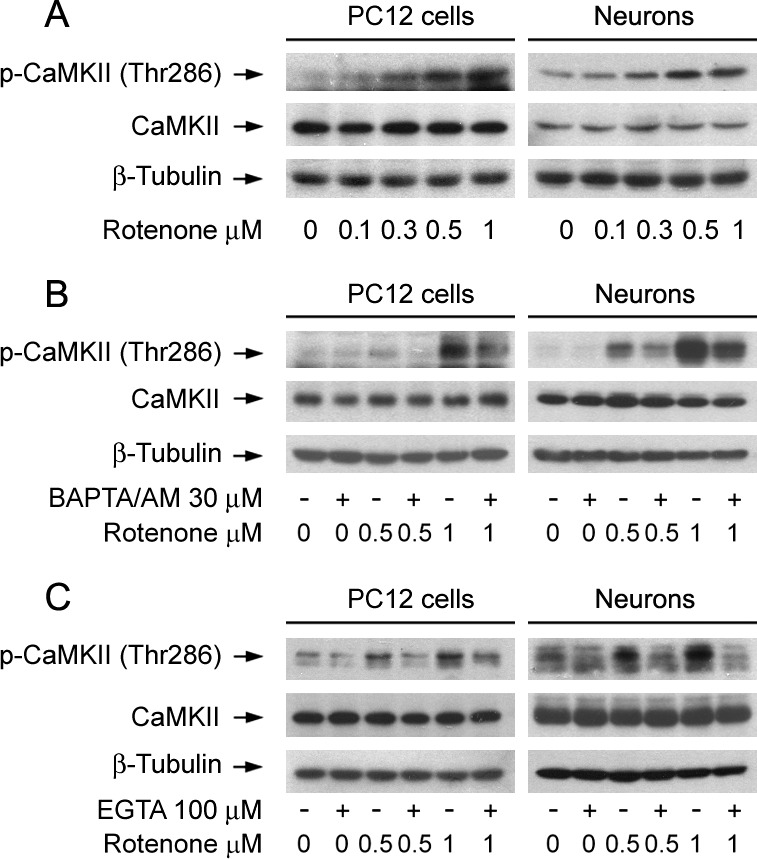
Rotenone triggers Ca^2+^-dependent CaMKII phosphorylation in neuronal cells PC12 cells and primary neurons were treated with rotenone (0-1 μM) for 24 h, or pretreated with/without BAPTA/AM (30 μM) or EGTA (100 μM) for 1 h and then exposed to rotenone (0.5 and 1 μM) for 24 h. Total cell lysates were subjected to Western blotting using indicated antibodies (A-C). The blots were probed for β-tubulin as a loading control. Similar results were observed in at least three independent experiments. **A.** Rotenone resulted in phospho-CaMKII increase in a concentration-dependent manner in the cells. **B.**, **C.** Pretreatment with BAPTA/AM and EGTA obviously inhibited rotenone-induced CaMKII phosphorylation in the cells, respectively.

CaMKII is a general integrator of Ca^2+^ signaling, which is activated by Ca^2+^/calmodulin (CaM) complex [41, 42]. To determine the role of CaMKII activity in rotenone-induced neuronal apoptosis, PC12 cells and primary neurons were exposed to 0-1 μM rotenone for 24 h. We found that rotenone induced phosphorylation of CaMKII in a concentration-dependent manner in the cells (Figure 3A). This was in agreement with decreased cell viability (Figure 1B) or increased apoptosis (Figure S1B, Figure 1C and 1D) in PC12 cells and primary neurons induced by rotenone exposure, suggesting that rotenone-induced neuronal apoptosis might involve induction of CaMKII phosphorylation.

Next, we studied whether rotenone induces phosphorylation of CaMKII is dependent on the level of [Ca^2+^]_i_. For this, PC12 cells and primary neurons were pre-incubated with/without BAPTA/AM (30 μM) or EGTA (100 μM) for 1 h, and then treated with/without rotenone (0.5 and 1 μM) for 24 h, respectively. As predicted, chelating [Ca^2+^]_i_ with BAPTA/AM or preventing [Ca^2+^]_i_ elevation using EGTA obviously inhibited rotenone-induced CaMKII phosphorylation in the cells (Figure 3B and 3C), indicating that rotenone-elevated [Ca^2+^]_i_ contributes to CaMKII phosphorylation.

**Figure 4:**
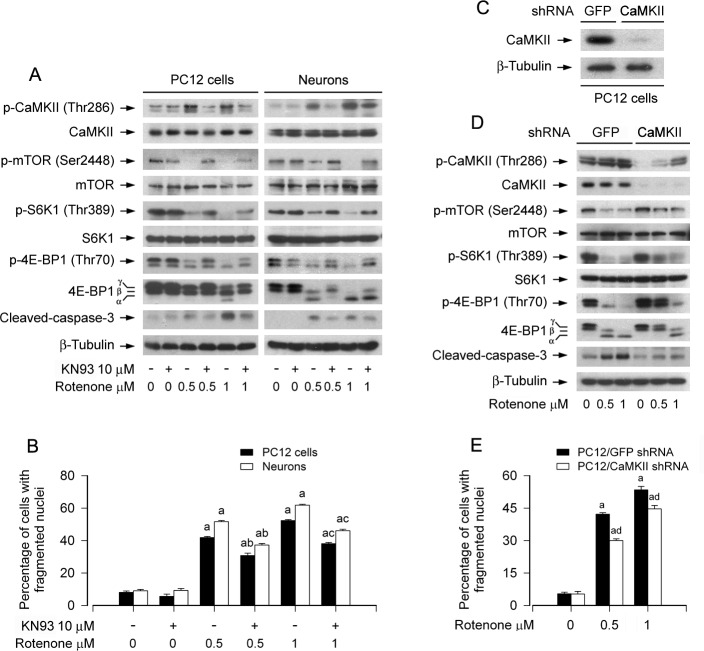
Pharmacological inhibition of CaMKII or downregulation of CaMKII partially prevents rotenone-induced inhibition of mTOR signaling and apoptosis in neuronal cells PC12 cells and primary neurons, or PC12 cells infected with lentiviral shRNA to CaMKII or GFP (as control), were treated with rotenone (0.5 and 1 μM) for 24 h, or pretreated with/without KN93 (10 μM) for 1 h and then exposed to rotenone (0.5 and 1 μM) for 24 h. Total cell lysates were subjected to Western blotting using indicated antibodies (A, C, D) The blots were probed for β-tubulin as a loading control. Similar results were observed in at least three independent experiments. Cell apoptosis was assayed using DAPI staining (B, E). **A**., **B**. Inhibition of CaMKII by KN93 partially prevented inhibition of mTOR, S6K and 4E-BP1 phosphorylation, cleavage of-caspase-3 (A) and apoptosis (B) in the cells induced by rotenone. **C.**-**E.** Lentiviral shRNA to CaMKII, but not to GFP, down-regulated CaMKII expression by ∼90% in PC12 cells (C), which obviously attenuated retonone-induced phosphorylation of CaMKII, and conferred partial resistance to rotenone-induced inhibition of mTOR signaling and activation of caspase-3 (D), as well as apoptosis (E) in the cells. Results are presented as mean ± SE (*n* = 5). ^a^*P* < 0.05, difference with control group; ^b^*P* < 0.05, difference with 0.5 μM rotenone group; ^c^*P* < 0.05, difference with 1 μM rotenone group; ^d^*P* < 0.05, CaMKII shRNA group versus GFP shRNA group.

To determine whether rotenone-induced phosphorylation of CaMKII contributes to inhibiting mTOR pathway and inducing apoptosis in neuronal cells, PC12 cells and primary neurons were pre-treated with CaMKII inhibitor KN93 (10 μM) for 1 h, and then exposed to rotenone (0.5 and 1 μM) for 24 h. We observed that rotenone-induced phosphorylation of CaMKII was obviously attenuated by KN93 in the cells (Figure 4A). Of importance, rotenone-inhibited phosphorylation of mTOR, S6K and 4E-BP1, as well as rotenone-activated caspase-3 were markedly prevented by KN93 (Figure 4A). Furthermore, KN93 also partially protected cell viability (Figure S3A), and significantly suppressed rotenone-induced nuclear fragmentation and condensation (Figure 4B).

To verify the role of CaMKII in rotenone suppression of mTOR pathway and neuronal apoptosis, the expression of CaMKIIα was silenced by RNA interference. As shown in Figure 4C, lentiviral shRNA to CaMKIIα, but not to GFP, downregulated CaMKII expression by ∼90% in PC12 cells. Silencing CaMKII obviously attenuated rotenone-induced phosphorylation of CaMKII (Figure 4D), and conferred partial resistance to rotenone-induced inhibition of phosphorylation of mTOR, S6K1 and 4E-BP1 (Figure 4D). Consistently, downregulation of CaMKII also attenuated rotenone-induced cleavage of caspase-3 (Figure 4D), as well as cell viability reduction (Figure S3B) and apoptosis (Figure 4E). The results clearly indicate that rotenone inhibits mTOR signaling pathway and neuronal apoptosis by elevating Ca^2+^-dependent CaMKII phosphorylation.

### Rotenone evokes Ca^2+^-CaMKII-dependent induction of H_2_O_2_ overproduction in neuronal cells

**Figure 5:**
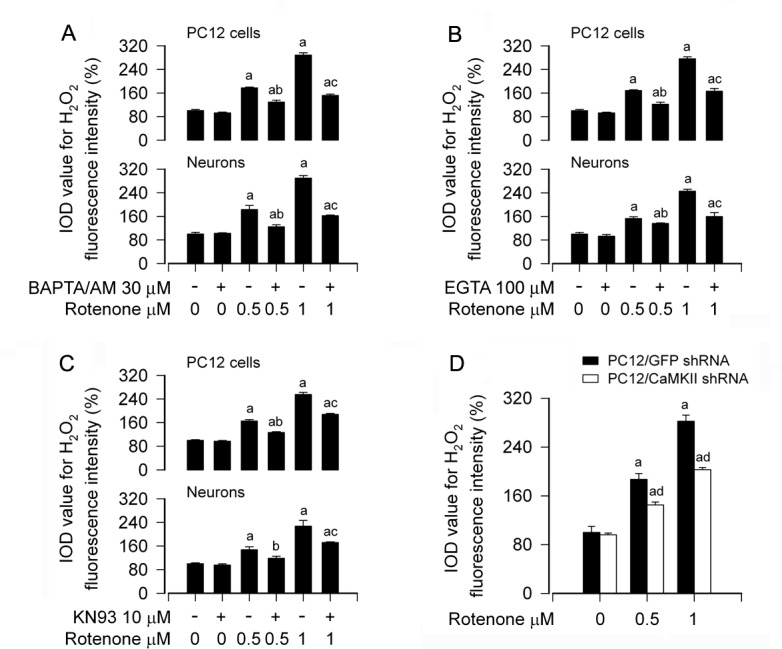
Rotenone evokes Ca^2+^-CaMKII-dependent induction of H_2_O_2_ overproduction in neuronal cells PC12 cells and primary neurons, or PC12 cells infected with lentiviral shRNA to CaMKII or GFP (as control), were treated with rotenone (0.5 and 1 μM) for 24 h, or pretreated with/without BAPTA/AM (30 μM), EGTA (100 μM) or KN93 (10 μM) for 1 h and then exposed to rotenone (0.5 and 1 μM) for 24 h, followed by intracellular H_2_O_2_ imaging using a peroxide-selective probe H_2_DCFDA. **A.**-**D.** H_2_O_2_ imaging was quantified, showing that BAPTA/AM (A), EGTA (B), KN93 (C) or downregulation of CaMKII (D) dramatically attenuated rotenone-induced H_2_O_2_ generation in the cells, respectively. Results are presented as mean ± SE (*n* = 5). ^a^*P* < 0.05, difference with control group; ^b^*P* < 0.05, difference with 0.5 μM rotenone group; ^c^*P* < 0.05, difference with 1 μM rotenone group; ^d^*P* < 0.05, CaMKII shRNA group versus GFP shRNA group.

Recently we have demonstrated that rotenone elicits H_2_O_2_ generation contributing to neuronal apoptosis [14]. In this study, we have noticed that rotenone-induced [Ca^2+^]_i_ elevation results in apoptosis of PC12 cells and primary neurons (Figures 1 and 2). Therefore, next we sought to test whether rotenone-elevated [Ca^2+^]_i_ contributes to H_2_O_2_ production and consequential apoptosis. To this end, PC12 cells and primary neurons were pretreated with/without BAPTA/AM (30 μM) or EGTA (100 μM) for 1 h, and then exposed to 0.5 and 1 μM rotenone for 24 h, followed by imaging H_2_O_2_ fluorescence using 2′7′-dichlorodihydrofluorescein diacetate (H_2_DCFDA), a peroxide-selective probe [43]. The results showed that rotenone induced a robust level of H_2_O_2_ (in green) in the cells (Figure S4), in consistence with our recent findings [14]. Interestingly, rotenone-induced H_2_O_2_ overproduction was profoundly attenuated by BAPTA/AM (Figure S4) or EGTA (data not shown) in the cells. Quantitative analyses of the fluorescence intensities of H_2_O_2_ generation inhibited by BAPTA/AM or EGTA are shown in Figure 5A and 5B. Similarly, pretreatment with CaMKII inhibitor KN93 also markedly diminished rotenone induction of H_2_O_2_ in PC12 cells and primary neurons (Figure 5C), implying that CaMKII is involved in rotenone-induced H_2_O_2_ generation in neuronal cells. This was further confirmed by the observation that silencing CaMKII obviously reduced rotenone-triggered H_2_O_2_ overproduction in PC12 cells (Figure 5D). These data strongly suggest that rotenone evokes Ca^2+^-CaMKII-dependent induction of H_2_O_2_ overproduction, leading to apoptosis in neuronal cells.

### Rotenone also induces mitochondrial H_2_O_2_-dependent [Ca^2+^]_i_ elevation activating CaMKII, leading to inhibition of mTOR pathway and neuronal apoptosis

**Figure 6:**
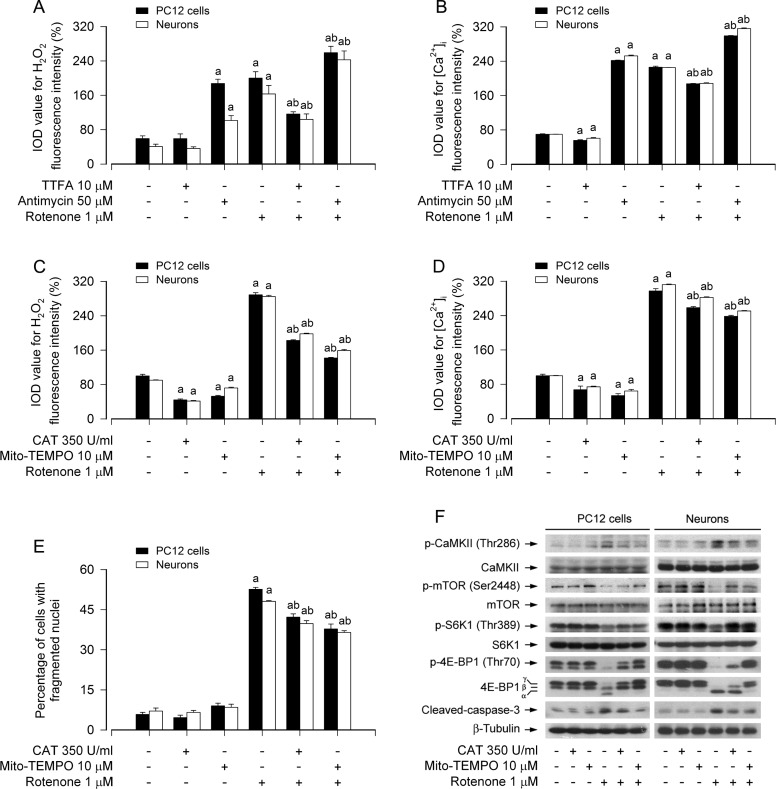
Rotenone induces mitochondrial H_2_O_2_-dependent [Ca^2+^]_i_ elevation activating CaMKII, leading to inhibition of mTOR pathway and neuronal apoptosis PC12 cells and primary neurons were treated with rotenone (1 μM) in the presence or absence of TTFA (10 μM) or antimycin A (50 μM) for 24 h, or pretreated with/without CAT (350 U/ml) or Mito-TEMPO (10 μM) for 1 h and then exposed to rotenone (1 μM) for 24 h. Fluorescence intensity for cell H_2_O_2_ (A, C). and [Ca^2+^]_i_ (B, D) was imaged and quantified using a peroxide-selective probe H_2_DCFDA and an intracellular Ca^2+^ indicator dye Fluo-3/AM, respectively. Cell apoptosis was assayed using DAPI staining (E). Total cell lysates were subjected to Western blotting using indicated antibodies (F). The blots were probed for β-tubulin as a loading control. Similar results were observed in at least three independent experiments. **A**. There existed an obvious decline in H_2_O_2_ fluorescence during co-treatment with rotenone and TTFA in the cells, whereas a further increase in H_2_O_2_ level in the cells exposed to rotenone in the presence of antimycin A. **B**. Administration of TTFA elicited a significant reduction of [Ca^2+^]_i_, yet antimycin A resulted in an enhancement of [Ca^2+^]_i_ triggered by rotenone. **C.**-**E.** Pretreatment with CAT or Mito-TEMPO significantly reduced intracellular H_2_O_2_ and [Ca^2+^]_i_ levels (C, D) and prevented apoptosis in the cells induced by rotenone (E). **F.** Pretreatment with CAT or Mito-TEMPO substantially attenuated rotenone-induced activation of CaMKII phosphorylation, inhibition of mTOR, S6K1/4E-BP1 phosphorylation, and cleaved-caspase-3 in the cells. Results are presented as mean ± SE (*n* = 5). ^a^*P* < 0.05, difference with control group; ^b^*P* < 0.05, difference with 0.5 μM rotenone group; ^c^*P* < 0.05, difference with 1 μM rotenone group.

Mitochondria are crucially involved in cellular Ca^2+^ and redox homeostasis and apoptosis induction [20, 44]. Because rotenone neurotoxicity is related to overproduction of mitochondrial ROS/H_2_O_2_ and disruption of Ca^2+^ homeostasis [14, 45], we next examined the association of rotenone-induced mitochondrial H_2_O_2_ generation with [Ca^2+^]_i_ elevation in neuronal cells. In line with our recent findings [14], when PC12 cells and primary neurons were treated with rotenone (1 μM) in the presence or absence of thenoyltrifluoroacetone (TTFA, 10 μM), a mitochondrial complex II ubiquinone site inhibitor, which blocks electron supply to ubiquinol and consequential limiting the formation of ubisemiquinone [46], for 24 h, we observed an obvious decline in H_2_O_2_ fluorescence during co-treatment with rotenone and TTFA in the cells (Figure 6A). In contrast, when cells were exposed to rotenone in the presence of antimycin A (50 μM), a mitochondrial complex III inhibitor, which increases the lifetime of ubisemiquinone [47], for 24 h, we noticed a further increase in H_2_O_2_ level in the cells (Figure 6A). Of note, treatment with TTFA also elicited a significant reduction of [Ca^2+^]_i_, yet antimycin A resulted in an enhancement of [Ca^2+^]_i_ triggered by rotenone (Figure 6B), implying that there exists mitochondrial H_2_O_2_-dependent [Ca^2+^]_i_ elevation in neuronal cells in response to rotenone.

To gain more insights into the event that rotenone induces mitochondrial H_2_O_2_-dependent [Ca^2+^]_i_ elevation leading to neuronal apoptosis, we extended our studies using catalase (CAT), a H_2_O_2_-scavenging enzyme, and Mito-TEMPO, a mitochondria-targeted antioxidant [48]. Pretreatment with CAT (350 U/ml) or Mito-TEMPO (10 μM) significantly reduced rotenone-induced H_2_O_2_ and [Ca^2+^]_i_ (Figure 6C and 6D), and prevented rotenone-induced apoptosis (Figure 6E) in PC12 cells and primary neurons. Consistently, CAT or Mito-TEMPO also diminished rotenone-induced cleavage of caspase-3 in the cells (Figure 6F). Furthermore, we found that pretreatment with CAT or Mito-TEMPO substantially attenuated rotenone-induced activation of CaMKII phosphorylation and inhibition of mTOR, S6K1/4E-BP1 phosphorylation in the cells (Figure 6F). Collectively, our findings demonstrate that rotenone induces mitochondrial H_2_O_2_-dependent [Ca^2+^]_i_ elevation, which activates CaMKII, leading to inhibition of mTOR pathway and consequential neuronal apoptosis.

### Ectopic expression of wild-type mTOR or constitutively active S6K1, or downregulation of 4E-BP1 partially prevents rotenone elevation of [Ca^2+^]_i_-mediated CaMKII phosphorylation and neuronal apoptosis

**Figure 7:**
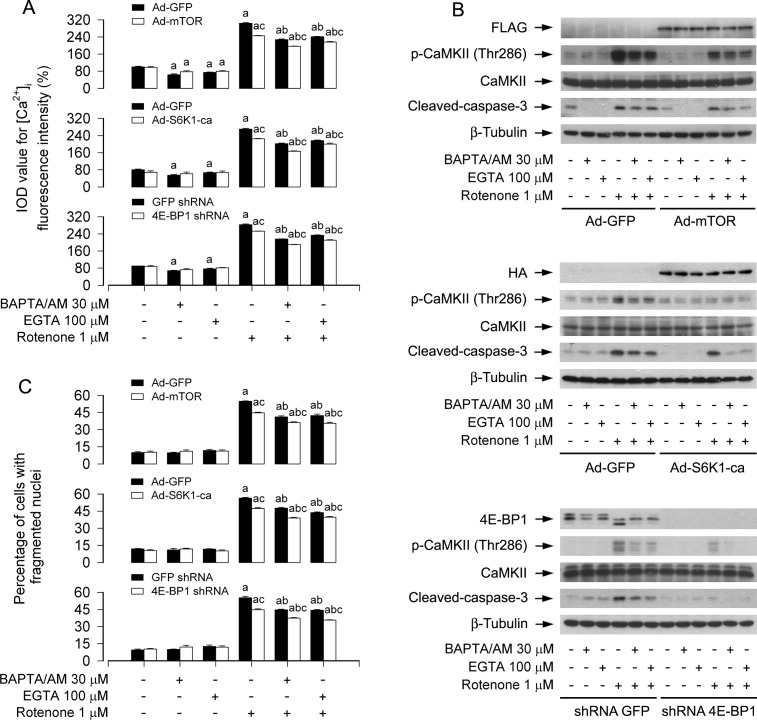
Ectopic expression of wild-type mTOR or constitutively active S6K1, or downregulation of 4E-BP1 reinforces BAPTA/AM's or EGTA's prevention of rotenone elevation of [Ca^2+^]_i_-mediated CaMKII phosphorylation and neuronal apoptosis PC12 cells infected with Ad-mTOR, Ad-S6K1-ca or Ad-GFP, or PC12 cells infected with lentiviral shRNA to 4E-BP1 or GFP, respectively, were pretreated with/without BAPTA/AM (30 μM) or EGTA (100 μM) for 1 h and then exposed to rotenone (1 μM) for 24 h. [Ca^2+^]_i_ fluorescence intensity was imaged and quantified using an intracellular Ca^2+^ indicator dye Fluo-3/AM (A). Total cell lysates were subjected to Western blotting using indicated antibodies (B). The blots were probed for β-tubulin as a loading control. Similar results were observed in at least three independent experiments. Cell apoptosis was assayed using DAPI staining (C). **A.**-**C.** Ectopic expression of wild-type mTOR or constitutively active S6K1, or silencing 4E-BP1 enhanced BAPTA/AM's or EGTA's prevention of rotenone-induced [Ca^2+^]_i_ elevation (A), CaMKII phosphorylation and cleaved-caspase-3 (B), as well as neuronal apoptosis (C). Results are presented as mean ± SE (*n* = 5). ^a^*P* < 0.05, difference *v*s control group; ^b^*P* < 0.05, difference with 1 μM rotenone group; ^c^*P* < 0.05, Ad-mTOR group or Ad-S6K1-ca group versus Ad-GFP group, or 4E-BP1 shRNA group versus GFP shRNA group.

Recently we have found that ectopic expression of wild-type mTOR (mTOR-wt) or constitutively active S6K1 (S6K1-ca), or downregulation of 4E-BP1 partially prevented rotenone-induced H_2_O_2_ and apoptosis in neuronal cells [14]. This prompted us to postulate that expression of mTOR-wt, S6K1-ca, or silencing 4E-BP1 may prevent rotenone-induced neuronal apoptosis by inhibiting mitochondrial H_2_O_2_ and blocking Ca^2+^ signaling. To test this concept, PC12 cells were infected with Ad-mTOR, Ad-S6K1-ca or Ad-GFP (as control), or with lentiviral shRNA to 4E-BP1 or GFP, and then exposed to rotenone (1 μM) for 24 h following pre-incubation with/without CAT (350 U/ml), Mito-TEMPO (10 μM), BAPTA/AM (30 μM) or EGTA (100 μM) for 1 h, respectively. As expected, expression of mTOR-wt or S6K1-ca, or silencing 4E-BP1 substantially blocked rotenone-induced elevation of [Ca^2+^]_i_ (Figure 7A and Figure S5A), phosphorylation of CaMKII (Figure 7B and Figure S5B), respectively. Consistently, rotenone-induced cleavage of caspase-3 was apparently attenuated by expression of mTOR-wt or S6K1-ca, or silencing 4E-BP1 (Figure 7B and Figure S5B). Furthermore, expression of mTOR-wt or S6K1-ca, or silencing 4E-BP1 also partially rescued PC12 cells from apoptosis induced by rotenone (Figure 7C and Figure S5C). Of importance, addition of CAT, Mito-TEMPO, BAPTA/AM or EGTA exhibited more inhibitory effects on rotenone-induced phosphorylation of CaMKII, inhibition of mTOR pathway, and induction of apoptosis in the cells infected with Ad-mTOR, Ad-S6K1-ca, or 4E-BP1 lentiviral shRNA than in Ad-GFP or GFP shRNA control group (Figure 7A-7C and Figure S5A-C). Collectively, our findings suggest that rotenone elevation of mitochondrial H_2_O_2_- and [Ca^2+^]_i_-mediated CaMKII phosphorylation inhibits mTOR-mediated S6K1 and 4E-BP1 pathways, leading to neuronal apoptosis; activation of mTOR pathway, in turn, can partially prevent rotenone from increasing mitochondrial H_2_O_2_ and [Ca^2+^]_i_, thereby attenuating rotenone-induced neuronal apoptosis.

## DISCUSSION

Ca^2+^ signaling is an important component of signal transduction pathways, which regulates a variety of physiological responses of neurons to neurotransmitters and neurotrophic factors, including cell survival responses [16, 49]. Several previous studies have shown that disturbances in cellular Ca^2+^ homeostasis trigger synaptic dysfunction, impaired plasticity, and neuronal degeneration such as PD, AD and HD [16-19]. Especially, prolonged change in Ca^2+^ distribution in cells elicits a set of biochemical cascades that lead to apoptosis [20, 21]. Rotenone, a broad-spectrum pesticide, has been reported to disrupt [Ca^2+^]_i_ homeostasis or induce [Ca^2+^]_i_ elevation, leading to apoptosis in neuronal cells, such as nigral dopaminergic neurons [45] and Neuro-2a cells [37]. Our group has recently demonstrated that rotenone inhibits mTOR-mediated S6K and 4E-BP1 pathways leading to neuronal apoptosis [14, 36]. However, it is not clear whether rotenone inhibits mTOR signaling in Ca^2+^ dependent manner. Here, we provide evidence that pretreatment with BAPTA/AM (an intracellular Ca^2+^ chelator) or EGTA (an extracellular Ca^2+^ chelator) significantly attenuated rotenone-induced elevation of [Ca^2+^]_i_ level, inhibition of mTOR pathway, and induction of apoptosis in PC12 cells and primary neurons (Figure 2A-2F). The findings indicate that rotenone inhibition of mTOR pathway, leading to neuronal apoptosis, is attributed to [Ca^2+^]_i_ elevation, which, at least, involves extracellular Ca^2+^ influx, in neuronal cells in response to rotenone.

CaMKII, a ubiquitously expressed multifunctional Ser/Thr kinase, regulates the survival of neuronal cells [33, 50-52]. It is activated upon binding of Ca^2+^/CaM complex [41, 42]. As CaMKII is a general integrator of Ca^2+^ signaling, we speculated that rotenone might inhibit mTOR pathway and induce neuronal apoptosis by elevating Ca^2+^-dependent CaMKII phosphorylation. In this study, we found that 24-h exposure to rotenone increased CaMKII phosphorylation concentration-dependently in PC12 cells and primary neurons. Chelating intracellular Ca^2+^ with BAPTA/AM obviously inhibited rotenone-induced CaMKII phosphorylation in the cells, suggesting a [Ca^2+^]_i_-dependent mechanism. To unveil whether CaMKII activity is essential for rotenone-induced inhibition of mTOR signaling contributing to neuronal apoptosis, KN93, a specific inhibitor of CaMKII [53], was used. We found that KN93 significantly blocked rotenone-induced CaMKII phosphorylation, and effectively prevented rotenone from inhibiting mTOR pathway and inducing apoptosis in PC12 cells and primary neurons (Figure 4A and 4B). Similar results were observed in the cells treated with lentiviral shRNA to CaMKII (Figure 4C-4E). These findings strongly support that CaMKII plays a bridging role between rotenone-induced elevation of [Ca^2+^]_i_ and inhibition of mTOR pathway, leading to neuronal apoptosis.

Mitochondria play a crucial role in cellular Ca^2+^ and redox homeostasis and apoptosis induction [20, 44]. In the mitochondria, the respiratory chain complexes I and III are the primary mitochondrial sources of univalent reduction of O_2_ into superoxide (O_2_^-^•) [20, 54]. Both SOD1 and SOD2 can catalyze the dismutation of O_2_^-^• into O_2_ and H_2_O_2_ [55]. Of note, H_2_O_2_ takes place mainly at the most vulnerable mitochondrial complex I [43]. For example, H_2_O_2_ in mitochondria *in situ* in isolated nerve terminals is sufficiently enhanced when mitochondrial complex I is inhibited at a small degree [9]. Rotenone elicits mitochondrial ROS/H_2_O_2_-dependent neuronal apoptosis via inhibiting mitochondrial complex I [3, 4, 14]. In the present study, we identified that rotenone evokes Ca^2+^-CaMKII-dependent induction of H_2_O_2_ overproduction in the mitochondria of neuronal cells. This is supported by the findings that pretreatment with BAPTA/AM, EGTA or KN93, or silencing CaMKII markedly reduced H_2_O_2_ overproduction of neuronal cells in response to rotenone (Figure S4 and Figure 5). Furthermore, TTFA (a mitochondria complex II inhibitor) or Mito-TEMPO (a mitochondria-targeted antioxidant) blocked rotenone-induced mitochondrial H_2_O_2_ production, preventing [Ca^2+^]_i_ elevation/CaMKII phosphorylation, mTOR inhibition and neuronal apoptosis (Figure 6). Collectively, the above findings suggest that rotenone induces mitochondrial H_2_O_2_, resulting in elevation of [Ca^2+^]_i_, and rotenone, in turn, evokes Ca^2+^-CaMKII-dependent overproduction of H_2_O_2_ in neuronal cells, inhibiting mTOR signaling and inducing neuronal apoptosis. Thus, there exists a crosstalk between Ca^2+^ signaling and mitochondrial H_2_O_2_, which is required for rotenone inhibition of mTOR signaling and induction of neuronal cell death.

In this study, we also noticed that expression of mTOR-wt or S6K1-ca, or silencing 4E-BP1 attenuated rotenone-induced elevation of [Ca^2+^]_i_, phosphorylation of CaMKII, and neuronal apoptosis (Figure 7 and Figure S5). The results suggest that on one hand, rotenone induces [Ca^2+^]_i_ and mitochondrial H_2_O_2_, resulting in elevation of inhibition of mTOR, and induction of neuronal apoptosis; on the other hand, activation of mTOR pathway can, in turn, partially prevent rotenone from increasing [Ca^2+^]_i_ and mitochondrial H_2_O_2_ and thus attenuating rotenone-induced neuronal apoptosis. Currently we have no clue regarding how mTOR regulates [Ca^2+^]_i_ and mitochondrial H_2_O_2_ in the cells in response to rotenone.

It has been known that many cell death stimuli alter the level of Ca^2+^ in the cytosol and the storage of Ca^2+^ in the intracellular organelles [33, 56]. [Ca^2+^]_i_ increase is usually triggered by Ca^2+^ mobilization from intracellular stores and/or Ca^2+^ entry from the extracellular space [15]. The endoplasmic reticulum (ER) is one of the major Ca^2+^ storage units in cells, and blockers of ER calcium channel, such as IP3 receptors, can effectively prevent Ca^2+^ release induced by various stimuli [15, 20]. The mitochondria-associated ER membrane (MAM), a structure for association of the mitochondria with the ER membrane, can integrate signal transduction pathways to regulate the communication and functional interactions between the ER and mitochondrion, especially in Ca^2+^ exchanges [20, 57]. Under pathological conditions, Ca^2+^ exchange between mitochondria and ER mediated by MAM is an important apoptotic control point [20]. Therefore, it remains to be determined whether rotenone disrupts Ca^2+^ homeostasis in the neuronal cells by increasing [Ca^2+^]_i_ elevation and/or decreasing [Ca^2+^]_i_ clearance. Understanding the underlying mechanisms may be helpful to uncover why overexpression of mTOR-wt or S6K1-ca or downregulation of 4E-BP1 suppresses rotenone-induced [Ca^2+^]_i_ elevation.

**Figure 8:**
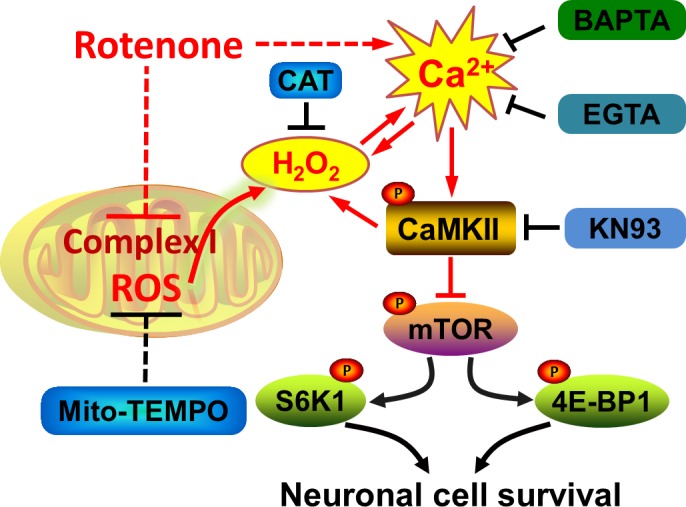
A schematic diagram showing how rotenone induces apoptosis by Ca^2+^/H_2_O_2_-dependent inhibition of mTOR pathway Rotenone-induced [Ca^2+^]_i_ elevation activates CaMKII, resulting in inhibition of mTOR-mediated S6K1 and 4E-BP1 pathways, which are crucial for cell survival. Rotenone elicits Ca^2+^-CaMKII-dependent H_2_O_2_ overproduction, and reversely rotenone also induces mitochondrial H_2_O_2_-dependent [Ca^2+^]_i_ elevation activating CaMKII, thereby inhibiting mTOR signaling and inducing neuronal apoptosis.

In summary, we have identified that rotenone-induced [Ca^2+^]_i_ elevation inhibits mTOR-mediated S6K1 and 4E-BP1 pathways contributing to neuronal apoptosis, through stimulating phosphorylation of CaMKII. Rotenone-induced extracellular Ca^2+^ influx at least plays an important role in contributing to neuronal apoptosis. Rotenone elicits Ca^2+^-CaMKII-dependent H_2_O_2_ overproduction, and reversely rotenone also induces mitochondrial H_2_O_2_-dependent [Ca^2+^]_i_ elevation activating CaMKII, thereby inhibiting mTOR signaling and reducing neuronal cell survival (i.e. inducing apoptosis) (Figure 8). Our findings highlight that the crosstalk between Ca^2+^ signaling and mitochondrial H_2_O_2_ is required for rotenone inhibition of mTOR pathway leading to neuronal apoptosis. The findings suggest that how to control over-elevation of intracellular Ca^2+^ and overproduction of mitochondrial H_2_O_2_ may be a new strategy to fight against the neurotoxicity of rotenone.

## MATERIALS AND METHODS

### Reagents

Rotenone, CAT, poly-D-lysine (PDL), H_2_DCFDA, 2′7′-dichlorofluorescein (DCF), protease inhibitor cocktail, TTFA, antimycin A, and DAPI, and EGTA were purchased from Sigma (St Louis, MO, USA). Dulbecco's modified Eagle medium (DMEM), 0.05% Trypsin-EDTA, NEUROBASAL^TM^ Media, and B27 Supplement were purchased from Invitrogen (Grand Island, NY, USA). Horse serum and fetal bovine serum (FBS) were supplied by Hyclone (Logan, UT, USA). Enhanced chemiluminescence solution was from Millipore (Billerica, MA, USA). CellTiter 96^®^ AQ_ueous_ One Solution Cell Proliferation Assay kit was from Promega (Madison, WI, USA). BAPTA/AM was purchased from Calbiochem (San Diego, CA, USA). Mito-TEMPO and KN93 were acquired from ALEXIS Biochemicals Corporation (San Diego, CA, USA). The following antibodies were used: phospho-CaMKII (Thr286), phospho-S6K1 (Thr389), phospho-4E-BP1 (Thr70), 4E-BP1, cleaved-caspase-3, PARP (Cell Signaling Technology, Beverly, MA, USA); S6K1, CaMKII (Santa Cruz Biotechnology, Santa Cruz, CA, USA); β-tubulin, phospho-mTOR (Ser2448), mTOR, HA, FLAG (Sigma); Goat anti-rabbit IgG-horseradish peroxidase (HRP), goat anti-mouse IgG-HRP, and rabbit anti-goat IgG-HRP (Pierce, Rockford, IL, USA). Other chemicals were purchased from local commercial sources and were of analytical grade.

### Cell lines, primary neurons and cultures

Rat pheochromocytoma (PC12) cell line (American Type Culture Collection, Manassas, VA, USA) was maintained in antibiotic-free DMEM supplemented with 10% horse serum and 5% FBS at 37°C in a humidified incubator containing 5% CO_2_. Primary cortical neurons were isolated from fetal mice at 16-18 days of gestation and cultured as described previously [35].

### Recombinant adenoviral constructs and infection of cells

The recombinant adenoviruses encoding N-terminal FLAG-tagged wild-type mTOR (Ad-mTOR-wt), HA-tagged constitutively active S6K1 (Ad-S6K1-ca), and the control virus encoding the green fluorescent protein (GFP) (Ad-GFP) were generated as described previously [35, 58]. The viruses were amplified, titrated and used as described [59].

### Lentiviral shRNA cloning, production, and infection

Lentiviral shRNAs to 4E-BP1, CaMKII and GFP (as control) were generated and used as described [33, 35]. For use, monolayer PC12 cells, when grown to about 70% confluence, were infected with above lentivirus-containing supernatant in the presence of 8 μg/ml polybrene and exposed to 2 μg/ml puromycin after 24 h of infection. In 5 days, cells were used for experiments.

### Analysis for cell viability

PC12 cells and primary neurons, seeded in a 96-well plate (1 × 10^4^ cells/well) pre-coated with PDL (0.2 μg/ml for PC12 cells; 10 μg/ml for primary neurons), were treated with 0-1 μM rotenone for 24 h, or with/without 0.5 and 1 μM rotenone for 24 h following pre-incubation with/without BAPTA/AM (30 μM), EGTA (100 μM), or KN93 (10 μM) for 1 h, with 5 replicates of each treatment. In some cases, PC12 cells, infected with lentiviral shRNA to CaMKII or GFP, respectively, were seeded in a 96-well plate (1 × 10^4^ cells/well). Next day, cells were exposed to 0.5 and 1 μM rotenone for 24 h. Subsequently, cell viability, after incubation with MTS reagent (one solution reagent) (20 μl/well) for 3 h, was evaluated by measuring the optical density (OD) at 490 nm using a Synergy^TM^ 2 Multi-function Microplate Reader (Bio-Tek Instruments, Inc. Winooski, Vermont, USA).

### DAPI and TUNEL staining

PC12 cells and primary neurons, PC12 cells infected with lentiviral shRNA to CaMKII, 4E-BP1 or GFP, or PC12 cells infected with Ad-mTOR, Ad-S6K1-ca or Ad-GFP, respectively, were seeded at a density of 5×10^5^ cells/well in a 6-well plate containing a PDL-coated glass coverslip per well, Next day, cells were treated with 0-1 μM rotenone for 24 h, or with/without 0.5 and/or 1 μM rotenone for 24 h following pre-incubation with/without BAPTA/AM (30 μM), EGTA (100 μM), KN93 (10 μM), CAT (350 U/ml) or Mito-TEMPO (10 μM) for 1 h, with 5 replicates of each treatment. Subsequently, the cells with fragmented and condensed nuclei were stained by adding DAPI (4 μg/ml in deionized water) as described [60]. Photographs for cell apoptosis were taken under a fluorescence microscope (Nikon 80i, Tokyo, Japan) equipped with a digital camera. In addition, for PC12 cells and primary neurons treated with 0-1 μM rotenone for 24 h, after DAPI staining, the following staining was performed by adding TUNEL reaction mixture (TdT enzyme solution and labeling solution) according to the manufacture's instructions of *In Situ* Cell Death Detection Kit^®^ (Roche, Mannheim, Germany). Finally, photographs were taken under a fluorescence microscope with a digital camera. For quantitative analysis of the fluorescence intensity using TUNEL staining, the integral optical density (IOD) was measured by Image-Pro Plus 6.0 software (Media Cybernetics Inc., Newburyport, MA, USA).

### Analysis for [Ca^2+^]_i_ imaging

PC12 cells and primary neurons, PC12 cells infected with lentiviral shRNA to 4E-BP1 or GFP, or PC12 cells infected with Ad-mTOR, Ad-S6K1-ca or Ad-GFP, respectively, were seeded at a density of 5×10^5^ cells/well in a 6-well plate containing a PDL-coated glass coverslip per well. Next day, cells were treated with 0-1 M rotenone for 24 h, with/without 1 μM rotenone in the presence or absence of TTFA (10 μM) or antimycin A (50 μM) for 24 h, or with/without 0.5 and/or 1 μM rotenone for 24 h following pre-incubation with/without BAPTA/AM (30 μM), EGTA (100 μM), CAT (350 U/ml) or Mito-TEMPO (10 μM) for 1 h. The cells were then loaded with 5 μM Fluo-3/AM for 40 min. Finally, all stained coverslips were rinsed twice with PBS, followed by imaging under a fluorescence microscope. IOD for quantitative analysis of the fluorescence intensity was analyzed by Image-Pro Plus 6.0 software as described above.

### Cell H_2_O_2_ imaging

Imaging intracellular H_2_O_2_ was recorded by using a nonfluorescent probe, H_2_DCFDA. This peroxide-selective dye can penetrate into the intracellular matrix of cells, where it is cleaved by intracellular esterases and oxidized by H_2_O_2_ to form fluorescent DCF [43]. In brief, PC12 and primary neurons, or PC12 cells infected with lentiviral shRNA to CaMKII or GFP, were seeded at a density of 5 × 10^5^ cells/well in a 6-well plate containing a glass coverslip per well. Next day, cells were treated with 0, 0.5, 1 μM rotenone for 24 h, or with/without 1 μM rotenone in the presence or absence of TTFA (10 μM) or antimycin A (50 μM) for 24 h, or with/without 0.5 and/or 1 μM rotenone for 24 h following pre-incubation with/without BAPTA/AM (30 μM), EGTA (100 μM), KN93 (10 μM), CAT (350 U/ml) or Mito-TEMPO (10 μM) for 1 h. The cells were then loaded with H_2_DCFDA (20 μM) for 1 h. Lastly, all stained coverslips were rinsed three times with PBS, followed by imaging under a fluorescence microscope. For quantitative analysis of the fluorescence intensity, IOD was measured by Image-Pro Plus 6.0 software as described above.

### Western blot analysis

PC12 cells and primary neurons, or PC12 cells infected with lentiviral shRNA to CaMKII, 4E-BP1 or GFP, or PC12 cells infected with Ad-mTOR, Ad-S6K1-ca or Ad-GFP, respectively, were treated with 0-1 μM rotenone for 24 h, or with/without 0.5 and/or 1 μM rotenone for 24 h following pre-incubation with/without BAPTA/AM (30 μM), EGTA (100 μM), KN93 (10 μM), CAT (350 U/ml) or Mito-TEMPO (10 μM) for 1 h. Afterwards, Western blotting was performed as described [35].

### Statistical analysis

Results were expressed as mean values ± standard error (mean ± SE). Student's *t*-test for non-paired replicates was used to identify statistically significant differences between treatment means. Group variability and interaction were compared using either one-way or two-way ANOVA followed by Bonferroni's post-tests to compare replicate means. Significance was accepted at *P* < 0.05.

## SUPPLEMENTARY MATERIAL FIGURES


