# Supplementary material for: Crosstalk between Ca2+ signaling and mitochondrial H2O2 is required for rotenone inhibition of mTOR signaling pathway leading to neuronal apoptosis
Source: Oncotarget. 2016 Feb 3;7(7):7534–49. doi: 10.18632/oncotarget.7183 (PMC4884936; doi:10.18632/oncotarget.7183)
Supplement: Supplementary file 1 [file oncotarget-07-7534-s001.pdf]

Crosstalk between Ca^{2+} signaling and mitochondrial H_2O_2 is required for rotenone inhibition of mTOR signaling pathway leading to neuronal apoptosis

Supplementary Material

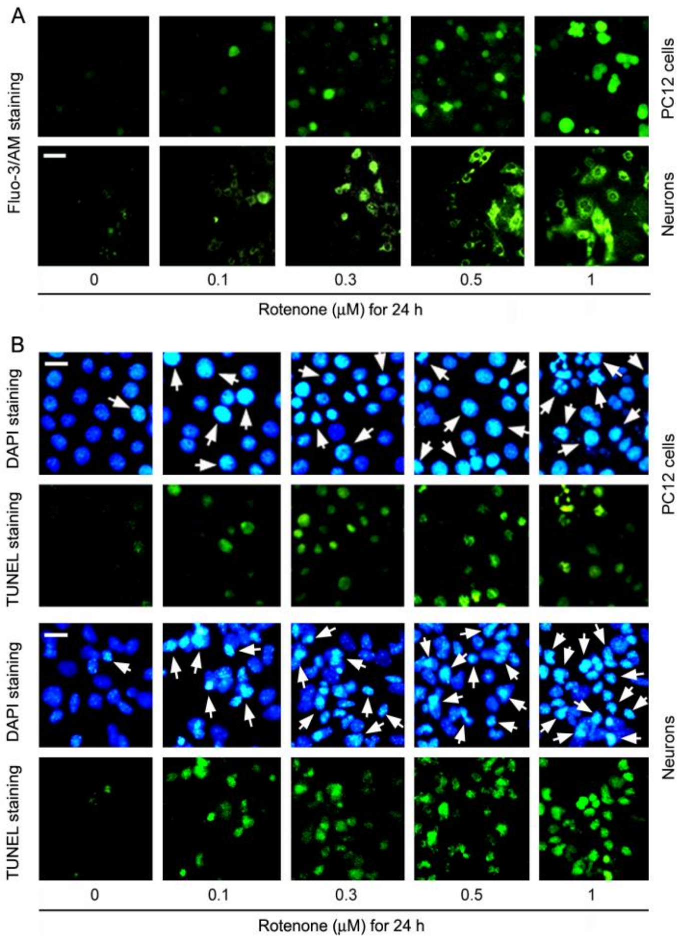

Supplementary Figure S1: Rotenone induces elevation of $[Ca^{2+}]_i$ imaging and apoptosis in neuronal cells. PC12 cells and primary neurons were treated with rotenone (0-1 μM) for 24 h. **A**. $[Ca^{2+}]_i$ imaging was labeled with an intracellular Ca^{2+} indicator dye Fluo-3/AM, showing that rotenone elicited strong $[Ca^{2+}]_i$ fluorescence (in green) in the cells. Scale bar: 50 μm. **B.** Cell apoptosis was assayed using DAPI and TUNEL staining. Shown are the cells with nuclear fragmentation and condensation (arrows) and TUNEL-positive cells (in green) with DNA brand breaks, respectively. Scale bar: 20 μm.

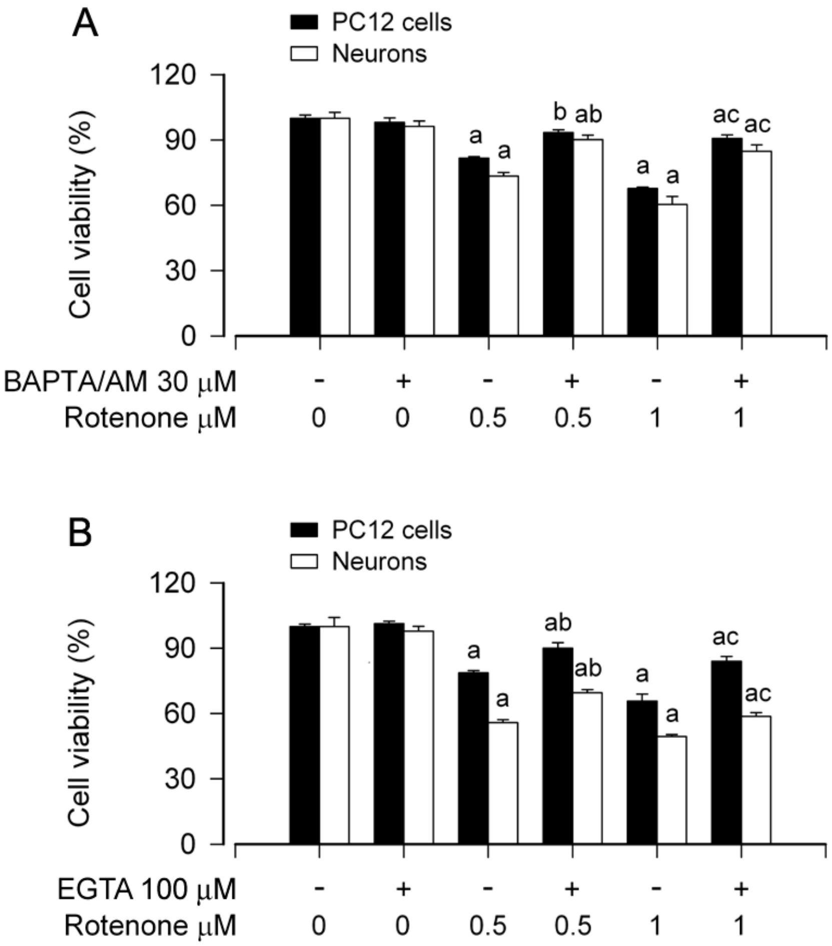

Supplementary Figure S2: Chelating [Ca^{2+}]$_i$ or preventing extracellular Ca^{2+} influx partially attenuates rotenone-induced viability reduction in neuronal cells. PC12 cells and primary neurons were pretreated with/without BAPTA/AM (30 μM) or EGTA (100 μM) for 1 h and then exposed to rotenone (0.5 and 1 μM) for 24 h, followed by cell viability assay using MTS. **A., B.** Chelating [Ca^{2+}]$_i$ with BAPTA/AM (A) or preventing extracellular Ca^{2+} influx using EGTA (B) obviously recused the cells from rotenone-induced viability reduction. Results are presented as mean ± SE (*n* = 5). $^{a}P<0.05$, difference with control group; $^{b}P<0.05$, difference with 0.5 μM rotenone group; $^{c}P<0.05$, difference with 1 μM rotenone group.

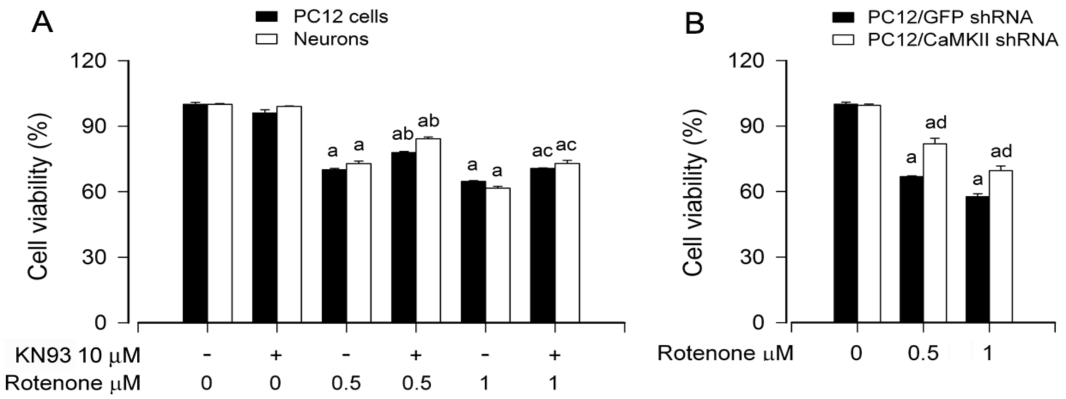

Supplementary Figure S3: Pharmacological inhibition of CaMKII or downregulation of CaMKII partially relieves rotenone-induced viability reduction in neuronal cells. PC12 cells and primary neurons, or PC12 cells infected with lentiviral shRNA to CaMKII or GFP (as control), were treated with rotenone (0.5 and 1 μM) for 24 h, or pretreated with/without KN93 (10 μM) for 1 h and then exposed to rotenone (0.5 and 1 μM) for 24 h. Cell viability was determined by the MTS assay. **A.** Inhibition of CaMKII by KN93 partially prevented loss of cell viability in the cells induced by rotenone. **B.** Lentiviral shRNA to CaMKII, but not to GFP, conferred partial resistance to rotenone-induced cell viability reduction in the cells. Results are presented as mean ± SE (*n* = 5). ^{a}P<0.05, difference with control group; ^{b}P<0.05, difference with 0.5 μM rotenone group; ^{c}P<0.05, difference with 1 μM rotenone group; ^{d}P<0.05, CaMKII shRNA group versus GFP shRNA group.

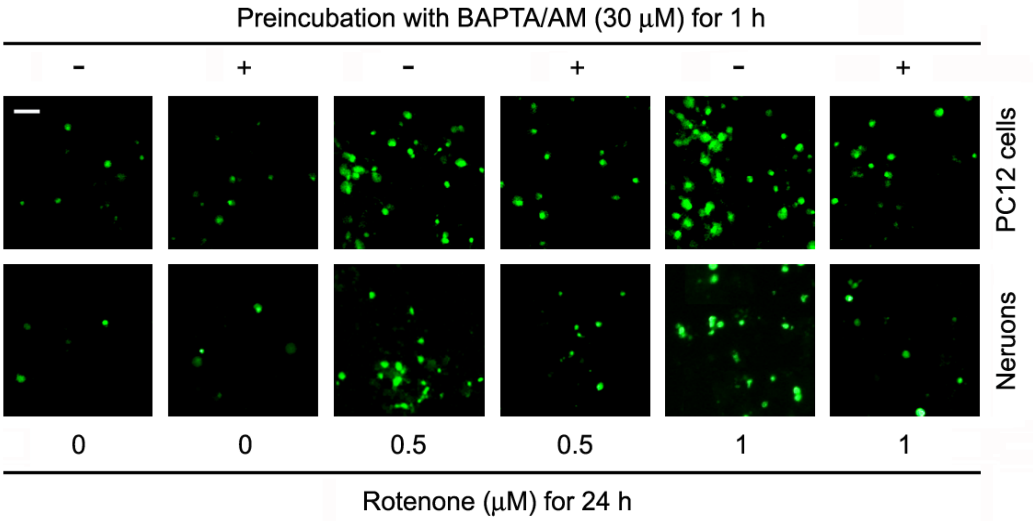

Supplementary Figure S4: Chelating $[Ca^{2+}]_i$ attenuates rotenone-elevated H_2O_2 imaging in neuronal cells. PC12 cells and primary neurons were pretreated with/without BAPTA/AM (30 µM) for 1 h and then exposed to rotenone (0.5 and 1 µM) for 24 h, followed by intracellular H_2O_2 imaging using a peroxide-selective probe H_2DCFDA. The cells with higher H_2O_2 fluorescence visualizations (in green) are profoundly attenuated by BAPTA/AM in the cells in response to rotenone. Scale bar: 20 µm.

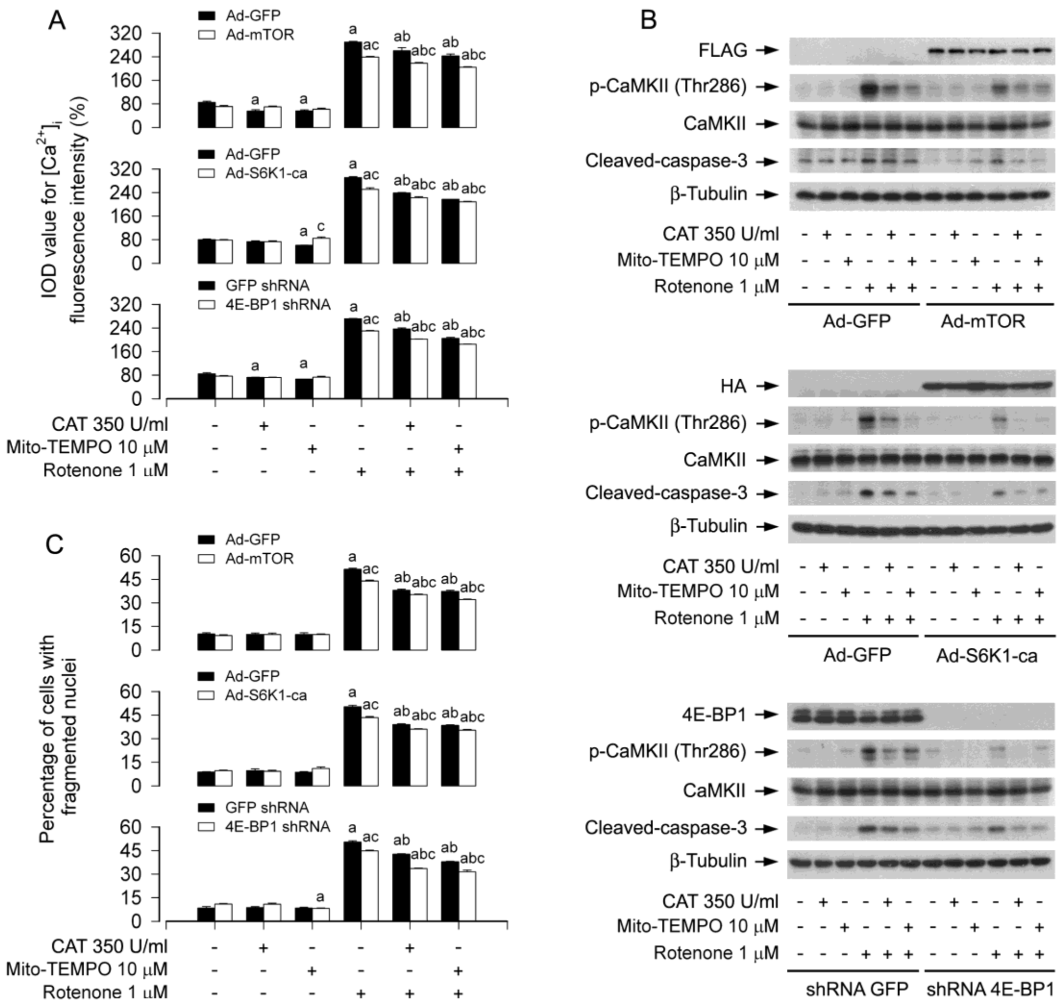

Supplementary Figure S5: Ectopic expression of wild-type mTOR or constitutively active S6K1, or downregulation of 4E-BP1 strengthens catalase's or Mito-TEMPO's inhibition of rotenone elevation of $[Ca^{2+}]_i$-mediated CaMKII phosphorylation and neuronal apoptosis. PC12 cells infected with Ad-mTOR, Ad-S6K1-ca or Ad-GFP, or PC12 cells infected with lentiviral shRNA to 4E-BP1 or GFP, respectively, were pretreated with/without CAT (350 U/ml) or Mito-TEMPO (10 μM) for 1 h and then exposed to rotenone (1 μM) for 24 h. $[Ca^{2+}]_i$ fluorescence intensity was imaged and quantified using an intracellular Ca^{2+} indicator dye Fluo-3/AM (A). Total cell lysates were subjected to Western blotting using indicated antibodies (B). The blots were probed for β-tubulin as a loading control. Similar

results were observed in at least three independent experiments. Cell apoptosis was assayed using DAPI staining (C). **A-C.** Ectopic expression of wild-type mTOR or constitutively active S6K1, or silencing 4E-BP1 strengthened the inhibitory effects of catalase or Mito-TEMPO on rotenone-induced $[Ca^{2+}]_i$ elevation (A), CaMKII phosphorylation and cleaved-caspase-3 (B), as well as neuronal apoptosis (C). Results are presented as mean ± SE (n = 5). $^{a}P<0.05$, difference *v*s control group; $^{b}P<0.05$, difference with 1 μM rotenone group; $^{c}P<0.05$, Ad-mTOR group or Ad-S6K1-ca group versus Ad-GFP group, or 4E-BP1 shRNA group versus GFP shRNA group.
